# Evaluation of the Blood Level of Adiponectin in Pregnant Adolescents

**DOI:** 10.1055/s-0041-1730288

**Published:** 2021-06-02

**Authors:** Elaine Cristina Rocha Pádua, Silvia Daher, Isa de Pádua Cintra Sampaio, Edward Araujo Júnior, Cristina Falbo Guazzelli

**Affiliations:** 1 Department of Obstetrics, Escola Paulista de Medicina, Universidade Federal de São Paulo, São Paulo-SP, Brazil; 2 Department of Pediatrics, Escola Paulista de Medicina, Universidade Federal de São Paulo, São Paulo-SP, Brazil

**Keywords:** adiponectin, adolescence, gestation, inflammation, adiponectina, adolescência, gestação, inflamação

## Abstract

**Objective** To evaluate serum levels of adiponectin in pregnant adolescents between 30 and 36 weeks of gestation.

**Method:** A prospective cross-sectional study enrolled 67 normal pregnant women between 30 and 36 weeks of gestation and eutrophic (body mass index [BMI]: 18.5–25 kg/m ^2^ ), of which 36 were adolescents (< 20 years old) and 31 adults (≥ 20 years old). Serum adiponectin levels were determined by enzyme-linked immunosorbent assay (ELISA). The *t-* student or Mann-Whitney tests were used for intergroup comparison.

**Results** Pregnant adolescents showed significantly higher serum adiponectin concentrations compared with pregnant adults ( *p* = 0.04). No differences were observed in adiponectin levels in younger pregnant adolescents (< 16 years old) compared with older pregnant adolescents (≥ 16 years old). Adiponectin values were divided into 3 subgroups: < 3,000 ng/mL, between 3,000 and 5,000 ng/mL, and > 5,000 ng/mL. Birthweight was significantly higher in women > 5,000 ng/mL when compared with < 3,000 ng/mL in the adolescent group. No association between pregestational adiponectin levels and BMI, gestational weight gain, and gestational age was observed; however, there was a positive relation with birthweight ( *p* = 0.0239).

**Conclusion** Serum adiponectin values in pregnant adolescents between 30 and 36 weeks of gestation were higher compared with pregnant adults; however, no differences between younger and older pregnant adolescents were observed.

## Resumo

**Objetivo** Avaliar os níveis séricos de adiponectina em gestantes adolescentes entre 30 e 36 semanas de gestação.

**Métodos** Estudo prospectivo e transversal incluindo 67 gestantes normais entre 30 a 36 semanas e eutróficas (índice de massa corporal [IMC]: 18,5–25 kg/m ^2^ ), sendo 36 adolescentes (< 20 anos) e 31 adultas (≥ 20 anos). Os níveis séricos de adiponectina foram avaliados por teste imunoenzimático (ELISA, na sigla em inglês). Para a comparação entre os grupos, utilizou-se os testes t-Student ou Mann-Whitney.

**Resultados** As gestantes adolescentes apresentaram significativamente maiores concentrações séricas de adiponectina do que as adultas ( *p* = 0,04). Não houve diferenças nos níveis de adiponectina quando comparadas as gestantes adolescentes precoces (< 16 anos) às tardias (≥ 16 anos). Os valores de adiponectina foram subdivididos em 3 grupos: < 3.000 ng/mL, entre 3.000 e 5.000 ng/mL e > 5.000 ng/mL. O peso do recém-nascido foi significantemente maior nas mulheres com > 5.000 ng/mL, quando comparadas as com < 3.000 ng/mL no grupo das adolescentes. Não foi observada associação entre os níveis de adiponectina e o IMC pré-gestacional, ganho de peso gestacional e a idade gestacional, porém houve relação positiva com o peso do recém-nascido ( *p* = 0,0239).

**Conclusão** Os valores séricos de adiponectina em gestantes adolescentes entre 30 e 36 semanas de gestação foram maiores do que os das gestantes adultas; contudo, sem diferenças entre gestantes adolescentes precoces e tardias.

## Introduction

Adiponectin is a polypeptide hormone abundantly produced and secreted by adipose tissue that regulates metabolism by interfering in insulin resistance in hepatic and cellular territories. 1 It stimulates glucose uptake by adipocytes and myocytes and directly activates adenosine monophosphate activated protein kinase (AMPK), acting as an insulin sensitizer. The main metabolic effects of adiponectin include glucose and lipid metabolism regulation through fatty acid oxidation stimulation, suppression of hepatic glucose production, and increased insulin sensitivity in liver and muscle tissue. 2 3 In contrast to other hormones secreted by the adipose tissue, its serum level decreases as adiposity increases and are negatively correlated with obesity, insulin resistance, and metabolic syndrome. 1 4 Furthermore, it presents other roles, presenting antihyperglycemic, antiatherogenic and anti-inflammatory properties. 5 6

Adiponectin is produced abundantly by adipose tissue and circulates at high concentration, in contrast to other adipokines. Although it is secreted by adipocytes, plasma adiponectin concentration is paradoxically lower in patients with type 2 diabetes mellitus, cardiovascular diseases, obesity, and in smokers. 7 Weight reduction in obese individuals is accompanied by an increase in plasma adiponectin concentration, suggesting that adipose tissue can exert a negative feedback on adiponectin production and secretion. 8 9

Serum adiponectin levels differ according to gender, being higher in women compared with men, even after matching for weight and body mass index (BMI). 10 In adolescents, no change in serum adiponectin concentration is observed between genders. However, studies showed that adiponectin levels, similar to what is observed in adults, are lower in obese adolescents and in pubescents. These values relate negatively to age and are significantly lower in puberty compared with the prepuberal period. Puberty is associated with decreased insulin sensitivity and changes in serum adiponectin concentrations. 11 12

Age effects in adiponectin production is still controversial, but many studies could observe differences in adiponectin levels between ages. It seems that adiponectin levels increase with advancing age, 13 14 15 16 17 and an experimental study indicated that estrogens have the ability to inhibit adiponectin production. 18 There are still few studies about adiponectin and puberty, but Lausten-Thomsen et al. 19 showed that, in adolescent women, adiponectin levels increase with increasing age and demonstrated how age- and sex-specific reference curves for adipokines are still necessary.

During pregnancy, there is a hypothesis that adiponectin may also play an important role in insulin resistance. 20 Lower concentrations of adiponectin have been consistently reported in patients with gestational diabetes mellitus (GDM) when compared with patients with a healthy pregnancy. 21 22

Since adiponectin has an essential role in insulin metabolism and that glucose and insulin are crucial for fetal growth, maternal adiponectin may play an important role in fetal development; however, the literature results about this association are still controversial. 23 24 25

The aim of the present study was to evaluate serum adiponectin concentration in pregnant adolescents between 30 and 36 weeks of gestation.

## Methods

A prospective cross-sectional study was conducted with eutrophic (BMI between 18.5 and 25 kg/m ^2^ ) pregnant adolescents (< 20 years old) and adults (≥ 20 years old), between 30 and 36 weeks of gestation. The subjects were selected from the Ambulatory of Prenatal Physiology of the Department of Obstetrics of Universidade Federal de São Paulo (UNIFESP, in the Portuguese acronym). The exclusion criteria were multiple pregnancies or chronic maternal diseases, such as arterial hypertension, pregestational diabetes mellitus or systemic lupus erythematosus. The present study was approved by UNIFESP's Ethics Committee (n ^o^ 1714/10), and all subjects signed the informed consent form.

A blood sample of 8 mL was drawn by venipuncture from the pregnant subject in a sterile and dry tube with separation gel (BD Diagnostics, Franklin Lakes, NJ, USA). The sample collected was centrifuged after clot retraction, and the obtained serum was aliquoted and stored in sterile microtube in a freezer at -80°C. Serum adiponectin levels were determined by enzyme-linked immunosorbent assay (ELISA) capture method using Quantikine-Human Adiponectin/Acrp30 DuoSet (R&D [R&D Systems Inc., Minneapolis, MN, USA]) commercial kit. This is an immunoenzymatic assay based on the sandwich technique performed according to the instructions of the manufacturer. Adiponectin sensitivity was 62.5 pg/mL.

Descriptive statistics with mean, median, minimum and maximum values and standard deviation (SD) was performed for all quantitative variables, and frequency analysis was performed for qualitative variables. The Kolmogorov-Smirnov or Shapiro-Wilk tests and Skewness and Kurtosis values were used to evaluate distribution for quantitative variables. Intergroup comparisons of quantitative variables were made using the *t-* Student test when distribution was normal and the Mann-Whitney test when distribution was non-normal.

Factorial analysis of variance (ANOVA) was used to compare the means of categorical variables by adiponectin concentration (ng/mL) and gestational group (adolescents and adults). When significant differences were observed by F statistic, the post-hoc Fisher LSD test was used to determine those differences.

Linear regression analysis was used to assess the association between adiponectin concentration and the other independent variables, with a 95% confidence interval (CI). Comparisons between adolescent and adult regressions were made using the *t-* Student test, and in the presence of significant differences, regression analyses were performed to define the better adjustment of curves.

To determine the influence of the variables study group, age, race, BMI, systolic and diastolic blood pressures, gestational age, pregnancy numbers, and birthweight on adiponectin concentration, a Stepwise Forward linear regression analysis was performed. Intergroup comparisons of qualitative variables were made using the chi-squared test, Fischer exact tests or G-tests.

The analyses were performed using GraphPad Prism 5.0 statistical package (GraphPad Software, San Diego, CA, USA). A statistical significance of *p* < 0.05 was adopted.

## Results

**Fig. 1:**
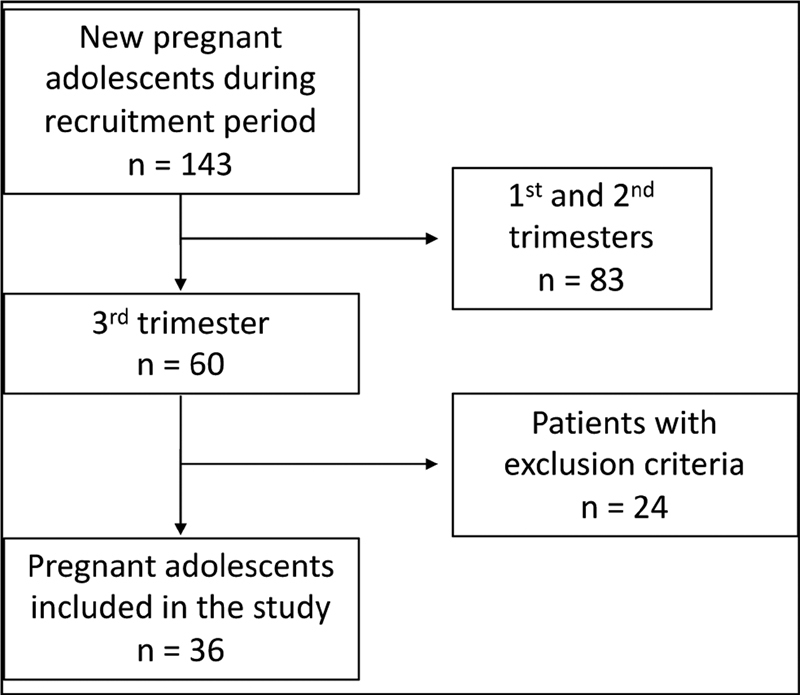
Flow chart of the selection of pregnant adolescents.

A total of 143 pregnant adolescents were followed-up, and blood samples were collected from 60 of them. In accordance with the exclusion criteria, 24 pregnant subjects were excluded, and, therefore, 36 were included ( Fig. 1 ). Thirty-one pregnant adults were selected, all with the same gestational period, absence of chronic maternal diseases and within the same BMI interval.

The age for pregnant adolescents varied from 13 to 19 years old, average of 16.53 years old, whereas in adults the age varied from 20 to 38 years old, average of 28.06 years old. Regarding marital status, the majority of the adolescents was single (63.9%), whereas adults were married (35.5%) or in a stable union (41.9%). Table 1 presents the sociodemographic characteristics of the study population.

**Table 1:** Sociodemographic, obstetric and perinatal characteristics of pregnant adolescents and adults

Variable	Adolescents *n * = 36	Adults *n* = 31	*p-value*
Age (years old) a	16.53 (1.98)	28.06 (4.99)	< 0.0001 *
Race b			0.19 **
White	13 (36.1%)	13 (41.9%)	
Multiracial	15 (41.7%)	16 (51.6%)	
Black	8 (22.2%)	2 (6.5%)	
Marital status b			0.003 **
Single	23 (63.9%)	6 (19.4%)	
Married	5 (13.9%)	11 (35.5%)	
Stable union	8 (22.2%)	13 (41.9%)	
Divorced	0 (0%)	1 (3.2%)	
Years of study (years) a	10.25 (1,96)	10.89 (2,13)	0.22 *
Salaried work b			< 0.0001 †
Yes	8 (22.2%)	22 (71.0%)	
No	28 (77.8%)	9 (29.0%)	
Smoking b			0.02 †
Yes	1 (2.8%)	7 (22.6%)	
No	35 (97.2%)	24 (78.4%)	
Alcohol abuse b			0.002 †
Yes	1 (2.8%)	10 (32.3%)	
No	35 (97.2%)	21 (67.7%)	
BMI (Kg/m ^2^ ) a	21.59 (2.20)	21.25 (1.62)	0.48 *
GA at collection (weeks) a	32.45 (1.58)	33.19 (1.51)	0.055 *
Number of pregnancies b			< 0.0001 †
1	35 (97.2%)	11 (35.5%)	
≥ 2	1 (2.8%)	20 (64.5%)	
Parturition b			< 0.0001 **
0	35 (97.2%)	14 (45.2%)	
1	1 (2.8%)	12 (38.7%)	
≥ 2	0 (0%)	5 (16.1%)	
Birthweight (grams) a	3103 (570.5)	3065 (245.0)	0.72 §

Abbreviations: BMI, body mass index; GA, gestational age.

a mean (standard deviation).

b absolute number (percentage).

* *t-* Student Test.

** Chi-squared Test.

† Fisher Test.

§ Welch adjusted *t-* Student Test.

**Fig. 2:**
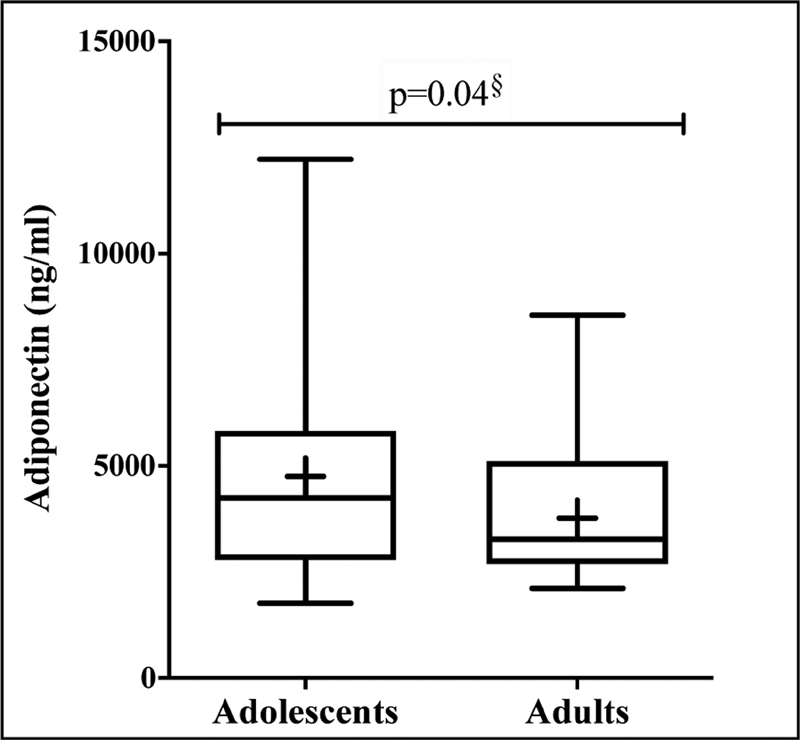
Serum adiponectin levels in pregnant adolescents ( *n* = 36) and adults ( *n* = 31) ^§^ Welch's adjusted *t-* Student Test.

In relation to the number of pregnancies and parturition, there was a significant intergroup difference, with fewer pregnancies ( *p* < 0.0001) and lower numbers of children ( *p* < 0.0001) in adolescents compared with adults. When we assessed gestational age at the time of collection and birthweight, no significant difference was observed between both groups ( Table 1 ). Serum adiponectin concentrations were significantly higher in pregnant adolescents compared with adults ( *p* = 0.04) ( Fig. 2 ).

In the younger adolescent group (< 16 years old), the age varied from 13 to 15 years old, with an average of 14.25 years old. In turn, in the older adolescent group (≥ 16 years old), the average age was 17.67 years old, varying from 16 to 19 years old. Older adolescents had more years of study (10.96 years) compared with the younger group (8.83 years). Regarding race, marital status and salaried work, there was no difference between groups ( Table 2 ).

**Table 2:** Sociodemographic characteristics of younger and older adolescents

Variable	Adolescents <16 *n* = 12	Adolescents ≥16 *n* = 24	*p-value*
Age (years old) a	14.25 (0.75)	17.67 (1.27)	< 0.0001 *
Race b			0.82 **
White	5 (41.7%)	8 (33.3%)	
Multiracial	5 (41.7%)	10 (41.7%)	
Black	2 (16.6%)	6 (25.0%)	
Marital status b			0.13 **
Single	6 (50.0%)	17 (70.8%)	
Married	1 (8.3%)	4 (16.7%)	
Stable union	5 (41.7%)	3 (12.5%)	
Years of study (years) a	8.83 (1.40)	10.96 (1.83)	0.001 *
Salaried work b			0.22 †
Yes	11 (91.7%) 1 (8.3%)	17 (70.8%) 7 (29.2%)	
No			
Smoking b			0.33 †
Yes	1 (8.3%)	0 (0%)	
No	11 (91.7%)	24 (100%)	
Alcohol abuse b			1.00 †
Yes	0 (0%)	1 (4.2%)	
No	12 (100%)	23 (95.8%)	
BMI (Kg/m ^2^ ) a	20.95 (1.97)	21.91 (2.28)	0.22 *

Abbreviation: BMI, body mass index.

a mean (standard deviation).

b absolute number (percentage).

* *t-* Student Test.

** Chi-square Test.

† Fisher Test.

Serum adiponectin concentrations showed no significant differences in both pregnant adolescent subgroups ( *p* = 0.57). We categorized adiponectin levels in 3 groups: < 3,000 ng/mL, between 3,000 ng/mL and 5,000 ng/mL, and > 5,000 ng/mL; and evaluated the intergroup difference and association with the independent variables. Factorial ANOVA was used to compare means (±SD) of categorical variables by adiponectin concentration (ng/mL) and gestational group (adolescents and adults). When significant differences were observed by F statistic, the post-hoc Fisher LSD test was used to determine those differences. It was possible to observe that adiponectin categories were related to birth weight, regardless of the pregnancy group ( Table 3 ).

**Table 3:** Influence of independent variables on adiponectin concentration (ng/mL) according to pregnancy group

	Adolescents			Adults			Statistic F		
	< 3,000 ( *n* = 10)	3,000–5,000 ( *n* = 12)	> 5,000 ( *n* = 14)	< 3,000 ( *n* = 12)	3,000–5000 ( *n* = 11)	> 5,000 ( *n* = 8)	F _(1, 66)_ ; *p-value* *	F _(2, 66)_ ; *p-value* **	F _(2, 66)_ ; *p-value* ***
Gestational age (days)	224.20 (11.16)	230.50 (11.67)	226.43 (10.54)	230.83 (9.76)	233.91 (14.09)	230.63 (9.16)	2.91; 0.09	1.08; 0.34	0.12; 0.88
Weight gain (kg)	13.45 (4.42)	13.47 (4.69)	13.34 (5.02)	10.85 (4.66)	10.47 (4.30)	11.94 (4.28)	4.15; 0.05	0.12; 0.89	0.17; 0.84
BMI (kg/m ^2^ )	21.26 (1.68)	21.53 (2.66)	21.89 (2.21)	20.83 (1.75)	21.16 (1.26)	22.00 (1.81)	0.22; 0.64	1.12; 0.33	0.12; 0.89
SBP (mm Hg)	104.00 (13.50)	105.83 (14.43)	107.14 (9.14)	90.00 (11.28)	102.73 (11.04) a §	95.00 (10.69) §	11.17; < 0.01	2.15; 0.13	1.38; 0.26
DBP (mm Hg)	65.00 (10.80)	70.00 (11.28)	69.29 (11.41)	54.17 (9.00) §	65.45 (11.28) a	58.75 (12.46) §	10.00; < 0.01	3.06; 0.05	0.58; 0.56
Birth weight (g)	2828.00 (564.88)	3037.58 (525.60)	3355.71 (539.18) a	2950.83 (210.00)	3056.36 (210.00)	3248.13 (310.11)	0.01; 0.91	5.01; 0.01	0.39; 0.68

Abbreviations: BMI, body mass index; DBP: diastolic blood pressure; SBP, systolic blood pressure.

* Adolescents and adults.

** Categories of adiponectin concentration.

*** Interaction between ‘adolescents and adults’ and ‘categories of adiponectin concentration’.

a Significant difference in adiponectin concentration < 3,000 ng/mL.

§ Significant difference for adolescents.

The association of serum adiponectin levels with the independent variables (gestational age, weight gain, BMI, systolic and diastolic blood pressures, and birthweight) was also evaluated using univariate linear regression. A positive relation between adiponectin levels and birthweight was observed in all assessed pregnant subjects ( *p* = 0.0239).

To determine the influence of variables on adiponectin concentration, a Stepwise Forward linear regression analysis was performed. In a first analysis of the main components that might influence adiponectin concentration in pregnant subjects, it was observed that some variables (race, pregnancy numbers, gestational age, and systolic and diastolic blood pressures) were not important to the model, considering the modification they produced together in the model (R ^2^ variation = 0.31%), absence of statistical significance (F _(9,57)_ = 1.56; *p* = 0.15), and lack of model adjustment (R ^2^ = 0.20; R ^2^_adjusted_ = 0.07).

A second analysis was performed. When just the “birthweight” variable was included, it resulted in a model statistically significant (F _(1,65)_ = 5.35; *p* = 0.02), but with poor association (R = 0.28). When “Birth weight” and “Age” variables were included, it resulted in a statistically significant model (F _(2,64)_ = 4.75; *p* = 0.01), but with a weak association (R = 0.36). Next, when the “birthweight,” “age” and “group (adolescents and adults)” variables were included, it resulted in a statistically significant model (F _(3,63)_ = 4.46; *p* = 0.01), but still with weak association (R = 0.42). Then, when the “birthweight,” “age,” “group (adolescents and adults)” and “BMI” variables were included, it resulted in a model with moderate association (R = 0.44), statistically significant (F _(4,62)_ = 3.74; *p* = 0.01), and with a better adjustment (R ^2^ = 0.20; R ^2^_adjusted_ = 0.15).

## Discussion

In the present study, it was observed that pregnant adolescents showed higher serum adiponectin levels compared with pregnant adults. Rasmussen-Torvik et al. 26 assessed serum adiponectin concentrations in male and female adolescents from 15 to 22 years old and observed higher levels in those with an average age of 15 years old compared with adolescents between 19 and 22 years old. In this study, the BMI was higher in older adolescents, from 19 to 22 years old, displaying greater abdominal circumference. Fifteen-year-old subjects showed a lower BMI, smaller abdominal circumference, and higher adiponectin concentration. The authors concluded that insulin sensitivity in younger adolescents was related to visceral fat, whereas adiponectin was associated with subcutaneous fat. 26 Among adult women, serum adiponectin levels tend to decrease as weight increases, in relation to an increase in adiposity, causing BMI changes. 16

Our results revealed higher adiponectin values in pregnant adolescents regardless of the age group. When younger adolescents (< 16 years old) were analyzed, no differences in serum adiponectin levels were observed when compared with older adolescents (≥ 16 years old). Both groups were highly heterogeneous regarding social, clinical, and obstetric characteristics, but with no differences in adiponectin levels between them. A possible explanation would be the small number of younger adolescents, in which higher adiponectin levels are expected. All adolescents had already experienced their respective menarche, so despite of separating younger from older adolescents, the hormonal variations probably responsible for the changes in blood adiponectin levels were not so evident in these groups.

Another important aspect of the present study was the racial balance between groups. Adiponectin values are strongly hereditary and are linked to genes that can be changed by race-dependent polymorphisms. Genetic load interferes with the prevalence of overweight or obesity in the studied population. 27 A significant association was observed between adiponectin values and single nucleotide polymorphism of the gene coding this protein. These changes are observed mainly in white women, but not among black women, reinforcing the difference observed between races. 28

Another factor that could interfere in adiponectin concentration would be weight gain during pregnancy and, again, there was no association in our study. Some studies revealed a negative correlation between adiponectin and maternal BMI; 29 30 however, in relation to our study, other studies did not attain the same results. 31 32

Adipokines not only influence maternal metabolism during pregnancy but may also affect fetal growth. 33 Our study showed a positive association between adiponectin levels and birthweight in all pregnant subjects evaluated. When the groups are studied separately, this association is demonstrated only in pregnant adolescents.

Pregnant adolescents with adiponectin levels > 5,000 ng/mL seem to give birth to more babies with adequate weight (∼ 3,000 g) compared with subjects with adiponectin values < 3,000 ng/mL that had insufficient average birthweight (< 3,000 g). Our findings are similar to those observed by Mazaki-Tovi et al., 34 who showed that maternal adiponectin levels are decreased when newborns present lower birthweight (< 2,999 g), described as insufficient.

The exact mechanism of how maternal adiponectin levels can affect birthweight still deserves more investigation. While there are studies that did not find this association, 25 35 other authors observed a negative association. 36 However, our result corroborates with another recent study that showed a positive association between maternal adiponectin and birthweight. 24 The association between maternal serum and umbilical cord adiponectin levels has been investigated, but the results are also conflicting. While some authors described an association between maternal and umbilical cord adiponectin, 23 others observed the opposite. 25 Aye et al. 37 proposed a mechanism by how adiponectin could affect birthweight, indicating that maternal adiponectin decreases placental insulin-signaling in the placenta, inhibiting fetal growth.

## Conclusion

In summary, we observed that serum adiponectin values were higher in pregnant adolescents than in pregnant adults; however, with no differences between younger and older pregnant adolescents. In addition to that, a significant difference in birthweight was observed when the categories of serum adiponectin concentration > 5,000 ng/mL and < 3,000 ng/mL were compared in pregnant adolescents.
